# Effects of COVID-19 convalescence on pregnancy outcomes in frozen-thawed embryo transfer: A retrospective cohort study

**DOI:** 10.1371/journal.pone.0326155

**Published:** 2025-07-01

**Authors:** Liwen Shen, Xiaoqin Pan, Yurong Zhu, Luping Liu

**Affiliations:** 1 Department of Center for Reproductive Medicine, Huzhou Maternity & Child Health Care Hospital, Huzhou, Zhejiang Province, China; 2 Nursing Department, Huzhou Maternity and Child Health Care Hospital, Huzhou, Zhejiang Province, China; Peking University Third Hospital, CHINA

## Abstract

In this study, we aimed to examine whether frozen-thawed embryo transfer during the recovery period after coronavirus disease can affect treatment outcomes. This population-based retrospective cohort study included patients who underwent frozen-thawed embryo transfer in the first cycle and did not have a history of coronavirus disease (n = 355, control group) or recovered from coronavirus disease within 6 months (n = 185) or 6–12 months (n = 230). Univariate analysis was performed to determine significant associations between the baseline variables, frozen-thawed embryo transfer cycle characteristics, clinical pregnancy rates, ongoing pregnancy rates, and pregnancy complication rates. Variables with significant associations in the univariate analysis were included in the multivariate logistic regression analysis to identify the effect of baseline characteristics, frozen-thawed embryo transfer cycle characteristics, and history of coronavirus disease on clinical pregnancy, ongoing pregnancy, and pregnancy complication rates. Patients who recovered from coronavirus disease within 6 months were more likely to experience complications during pregnancy than control group patients (33.7% vs. 20.3%, *p* = 0.001). Multivariate logistic regression analysis confirmed that a history of coronavirus disease within 6 months (odds ratio: 2.34, 95% confidence interval: 1.93–4.58) was a risk factor for pregnancy complications; however, a history of coronavirus disease was not a risk factor for clinical pregnancy rate, ongoing pregnancy rate, human chorionic gonadotrophin positivity rate, abortion rate, or live birth rate. Frozen-thawed embryo transfer can be conventionally performed during the recovery period after coronavirus disease; however, enhanced monitoring and follow-up during pregnancy are necessary to ensure the safety of the entire pregnancy and delivery process.

## Introduction

Since its outbreak in Wuhan, the novel coronavirus disease (COVID-19) epidemic has caused enormous losses in China and worldwide, including economic regression and health damage, and many couples have temporarily suspended their plans for pregnancy. Besides the respiratory and immune system stress reactions, severe acute respiratory syndrome-related coronavirus (SARS-CoV-2) can also be expressed in the endometrium and follicular fluid of the ovaries, adversely affecting female reproductive function [1–3].

Although some studies have reported that COVID-19 does not have a negative impact on the treatment outcomes of assisted reproductive technology (ART) cycles, such as egg retrieval rate, oocyte maturation rate, fertilization rate, number of high-quality embryos, and clinical pregnancy rates in ART cycles [4–7], the findings from these studies are mostly observed in the treatment outcomes after fresh embryo transfer. Since the widespread epidemic of COVID-19 in China at the end of 2022, the majority of patients undergoing in vitro fertilization-embryo transfers have opted to wait for frozen-thawed embryo transfers (FET) after recovery from COVID-19 for safety. However, evidence on the safety of FET in Chinese patients after recovery from COVID-19 is lacking.

Given the lack of SARS-CoV-2 infection-related FET research, this study aimed to investigate the effects of FET performed during the recovery period after SARS-CoV-2 infection on treatment outcomes. The early and late pregnancy outcomes of patients who underwent FET before and after the COVID-19 epidemic at a reproductive medicine center were compared. The authors believe that clarifying the impact of post-infection recovery time on pregnancy outcomes will facilitate informed decision-making regarding whether frozen embryos should be immediately thawed or if sufficient time should be allowed before transplantation.

## Materials and methods

### Study design and ethical approval

This was a single-center, retrospective cohort study. The study protocol was approved by the Ethics Committee of Huzhou Maternal and Child Health Care Hospital (No. 2024-J-028, March 25, 2024). The data collected for the study were routinely registered during ART, and the ethics committee waived the requirement for written informed consent owing to the retrospective nature of the study. Clinical outcome data were collected until March 31, 2024, which was the last follow-up date. All data were accessed from April 5, 2024, to April 10, 2024, for research purposes. The relevant study data were analyzed and interpreted by the authors responsible for reviewing the manuscript, confirming the completeness and accuracy of the data and ensuring strict adherence to the study protocol. The study was conducted in accordance with the relevant principles of the Declaration of Helsinki, and the results were reported in accordance with the Strengthening of the Reporting of Observational Studies in Epidemiology.

### Participants and study setting

This retrospective study was conducted at the Center for Reproductive Medicine of Huzhou Maternal and Child Health Care Hospital. Considering that the COVID-19 epidemic outbreak occurred at the end of 2019, patients without SARS-CoV-2 infection who underwent FET in 2019 were included in the control group (n = 355), and those who underwent FET in 2023 and had a COVID-19 history of < 6 months (n = 185) or > 6 months (n = 230) were included in the study group.

### Study procedures

Patients were asked about their history of COVID-19 when the FET archive was established. At the end of 2022, owing to the pandemic in the Huzhou area, most people experienced COVID-19. Patients who received a positive nasal or pharyngeal test for SARS-CoV-2 nucleic acid or who used an antigen test kit issued free of charge by the government were defined as having a history of COVID-19. The disappearance of COVID-19-related symptoms was identified as recovery. To clarify whether there were differences in the duration between infection and recovery from COVID-19, the study group was categorized into two groups: patients who recovered from COVID-19 within 6 months and those who recovered within 6–12 months. Patients were excluded if they underwent sequential transfers or had no embryo to use because the embryo was downgraded after thawing. For repeated transfers, we included FET cases from the first cycle. During the COVID-19 pandemic, many people were infected with the novel coronavirus, so we cannot guarantee that all embryos were obtained before the couples were exposed. However, all transferred embryos were vitrified-frozen and obtained in an environment free of COVID-19, ensuring that the clinicians, embryologists, caregivers, and couples involved were not infected at the time.

All thawed embryos were obtained from embryos frozen by vitrification. They were first transferred into vitrification solution (VS) 1 for 8 min. Thereafter, they were transferred into VS2 for 1 min. The embryos were then loaded into freezing carriers within 2 min. The carriers with embryos were promptly placed into liquid nitrogen, loaded into plastic sleeves, and thereafter into stents, and placed in liquid nitrogen storage tanks at −196°C after double-checking for accuracy.

Before FET, endometrial preparation was performed in three ways. (1) Hormone replacement cycle: on the second day of the menstrual cycle, 3 mg of estradiol valerate (Bayer HealthCare Ltd. Guangzhou Branch) was administered twice daily for 7 days; then, 4 mg of the same medication was taken twice daily for the next 7 days. When the endometrial thickness was ≥ 7 mm, 40 mg of progesterone (Zhejiang Xianju Pharmaceutical) was injected twice daily to support the transformation of the endometrial lining. The cleavage-stage embryo or blastocyst was transferred 3 or 5 days later, respectively. (2) Natural cycle: from the 10th to the 12th day of the menstrual cycle, the ovarian follicles were monitored. When the follicular diameter was ≥ 16 mm and endometrial thickness was ≥ 7 mm, the serum concentrations of luteinizing hormone (LH), estradiol (E2), and progesterone (P) were assessed. When LH was < 20 IU/L, we injected 10000U human chorionic gonadotrophin (HCG, Livzon Pharmaceutical, Guangdong, Zhuhai) at 9 PM. Three days later, 20 mg of dydrogesterone (Abbott Healthcare Products, the Netherlands, Olst) was administered twice daily. The cleavage-stage embryo or blastocyst was transferred 2 or 4 days later, respectively. When LH was ≥ 20 IU/L, 10000 U HCG was injected at 2 PM. Two days later, 20 mg of dydrogesterone was administered twice daily. The cleavage-stage embryo or blastocyst was transferred 2 or 4 days later, respectively. (3) Stimulated cycle: from the third day of the menstrual cycle, 2.5–5 mg of letrozole (Jiangsu Hengrui) was administered daily for 7 days. For the monitoring of ovarian follicles, we injected human menopausal gonadotrophin (Livzon Pharmaceutical) as necessary. When the follicular diameter was ≥ 16 mm and endometrial thickness was ≥ 7 mm, the serum concentrations of LH, E2, and P were assessed. When LH was < 20 IU/L, we injected 10000U HCG at 9 PM. Three days later, 20 mg of dydrogesterone was administered twice daily. The cleavage-stage embryo or blastocyst was transferred 3 or 5 days later, respectively. When LH was ≥ 20 IU/L, 10000 U HCG was injected at 2 PM. Two days later, 20 mg of dydrogesterone was administered twice daily. The cleavage-stage embryo or blastocyst was transferred 2 or 4 days later, respectively.

All thawed embryos were cryopreserved in vitrification and, on the day of frozen embryo transfer, thawed as follows: embryos were quickly washed with two different thawing solutions (TS1 and TS2) at 37°C and then transferred to TS3 and TS4 at 25 ± 2°C. Finally, the embryos were carefully rinsed in a well-balanced G2-PLUS dish and incubated in 6% CO2 and 5% O2 at 37°C for 1–2 h before transfer. The embryos were then transferred to EmbryoGlue transfer dishes 10–30 min before transfer. All reagents used for the vitrification and thawing of embryos were sourced from KITAZA-TO, Japan.

Patients were routinely asked to return to the hospital for follow-up after transplantation. After confirming biochemical pregnancy with an HCG test 14 days after transplantation, the pregnancy status of the patients was monitored until the pregnancy was terminated or interrupted. All data were registered in the Reproductive Management System and were only accessible to the staff of the Center for Reproductive Medicine to avoid data leakage.

### Study outcomes

The primary outcomes were clinical pregnancy, ongoing pregnancy, and pregnancy complication rates, whereas the secondary outcomes were HCG positivity, abortion, and live birth rates.

### Study definitions

Clinical pregnancy was defined as the presence of a pregnancy sac, which was detected using ultrasound examination 30 days after transplantation. Ongoing pregnancy was defined as a pregnancy that lasted for more than 20 weeks with a viable fetus. Complications during pregnancy included gestational hypertension, gestational diabetes, intrahepatic cholestasis, premature rupture of membranes, and placenta previa.

### Covariates

We adjusted for potential covariates known to significantly impact outcomes in frozen – thawed embryo transfer based on previous literature. These covariates included age, body mass index, follicle-stimulating hormone level, infertility type, infertility cause, fertilization method, endometrial preparation program, number of embryos transferred, and developmental stage of the embryos transferred. Detailed definitions of these covariates are provided in S1 Table.

### Statistical analyses

Descriptive statistical analysis was conducted based on normality tests (Kolmogrov- Smirnov), which revealed that the data followed a non-normal distribution. Therefore, non-parametric tests were used for the comparisons. Continuous variables are presented as the median and upper and lower quartiles [M (P25, P75)], which were compared using non-parametric Mann–Whitney U tests. Categorical variables are presented as proportions (percentages) and were compared using the chi-squared or Fisher exact test, as appropriate, to determine significant associations between the baseline variables and FET cycle characteristics. Variables that showed significant associations in the univariate analysis were included in the multivariate logistic regression analysis to identify the effect of the baseline and FET cycle characteristics on clinical pregnancy, ongoing pregnancy, and pregnancy complication rates. The Hosmer-Lemeshow goodness-of-fit statistic was used to assess the model. Age was stratified using 35 years as the threshold to improve the fitness of the model. All tests were two-tailed, and statistical significance was set at *p < *0.05. The analysis was performed using IBM SPSS Statistics for Mac, version 27.0.

## Results

**Fig 1:**
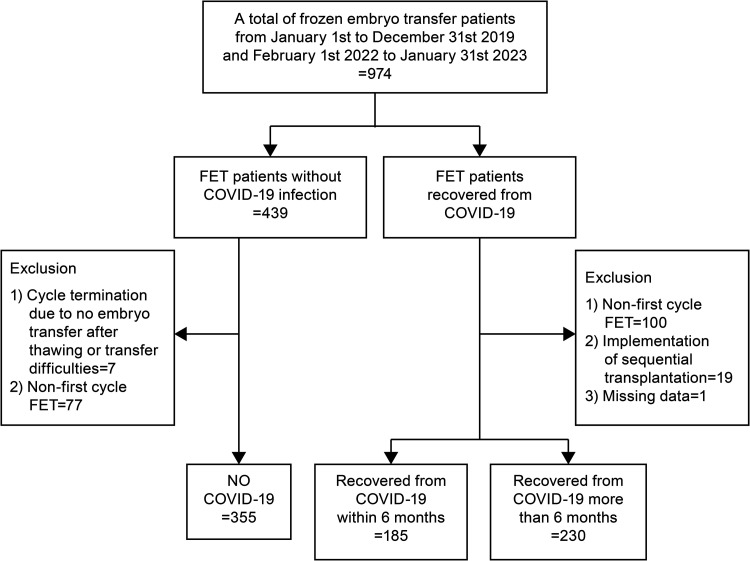
Study flowchart. Study flowchart showing the number of participants involved at each stage of the study. FET, frozen-thawed embryo transfer; COVID-19, coronavirus disease.

After data collection, 760 patients who underwent FET were included in the study. The study population consisted of three groups: 355 patients were uninfected with COVID-19 and had their cycles, 185 patients had their cycles during the recovery period within 6 months of contracting COVID-19, and 230 patients had their cycles during the recovery period within 6–12 months of contracting COVID-19 (Fig 1).

The clinical and FET cycle characteristics, treatment outcomes, and pregnancy outcomes of the groups are shown in Table 1. The mean age, body mass index, and basal follicle-stimulating hormone levels were not significantly different in the pairwise comparisons. A significantly higher proportion of patients with secondary infertility was noted in the group that recovered from COVID-19 within 6–12 months than in the control group (58.7% vs. 47.6%, *p* = 0.009). The investigation yielded a statistically significant difference in pregnancy complication rates between the control group and those that recovered from COVID-19 within 6–12 months (20.3% vs. 33.7%, *P* = 0.013). S2 Table shows the type and number of pregnancy complications in each group

**Table 1:** Characteristics of patients with and without a history of COVID-19 who underwent FET.

	(1) Patients without COVID-19 (N = 355)	Patients with COVID-19		*p*-value (1) vs. (2)	*p-*value (1) vs. (3)
		(2) Recovery within 6 months (N = 185)	(3) Recovery within 6–12 months (N = 230)		
**Clinical characteristic**					
Age, y	30 (28, 34)	31 (28, 35)	32 (28, 35)	0.072	0.013
BMI, kg/m^2^	22.1(20, 24.7)	21.9 (20.5, 24.1)	22.6 (20.68, 25.3)	0.090	0.056
FSH, IU/L	6.4 (5.6, 7.5)	6.5 (5.4, 7.4)	6.2 (5.3, 7.4)	0.863	0.138
Infertility type, n (%)					
Primary infertility	186 (52.4)	88 (47.6)	95 (41.3)	0.287	0.009
Secondary infertility	169 (47.6)	97 (52.4)	135 (58.7)		
Infertility cause, n (%)					
Female factor	274 (77.1)	136 (73.5)	189 (82.2)	0.224	0.323
Male factor	35 (9.9)	22 (11.9)	15 (6.5)		
Both factors	35 (9.9)	15 (8.1)	17 (7.4)		
Unexplained infertility	11 (3.1)	12 (6.5)	9 (3.9)		
**FET cycle characteristic**					
Fertilization method, n (%)					
IVF	271 (76.3)	144 (77.8)	197 (85.7)	0.823	0.016
ICSI	71 (20.0)	136 (19.5)	30 (13)		
IVF+ICSI	13 (3.7)	5 (2.7)	3 (1.3)		
Endometrial preparation program					
Stimulated cycle	27 (7.6)	4 (2.2)	9 (3.9)	<0.001	<0.001
Natural cycle	109 (30.7)	31 (16.7)	35 (15.2)		
HRT cycle	219 (61.7)	150 (81.1)	186 (80.9)		
Developmental stage of the embryos transferred, n (%)					
Cleavage stage	201 (56.6)	39 (21.1)	47 (20.4)	<0.001	<0.001
Blastocyst stage	154 (43.4)	146 (78.9)	183 (79.6)		
Number of embryos transferred, n (%)					
1	112 (31.5)	129 (69.7)	179 (77.8)	<0.001	<0.001
2	243 (68.5)	56 (30.3)	51 (22.2)		
**Reproductive outcome**					
Positive HCG rate (%)	214/355 (60.3)	112/185 (60.5)	143/230 (62.2)	0.953	0.647
Clinical pregnancy rate (%)	187/355 (52.7)	98/185 (53.0)	123/230 (53.5)	0.948	0.849
Abortion rate (%)	30/187 (16.0)	21/98 (21.4)	NA	0.407	NA
Ongoing pregnancy rate (%)	161/355 (45.4)	77/185 (41.8)	NA	0.260	NA
Pregnancy complications rate (%)	38/187 (20.3)	33/98 (33.7)	NA	0.013	NA
Live birth rate (%)	157/355 (44.2)	76/185 (41.1)	NA	0.484	NA

Data are presented as median (interquartile range) or n (%)

COVID-19, coronavirus disease; FET, frozen-thawed embryo transfer; SD, standard deviation; BMI, body mass index; FSH, follicle-stimulating hormone; IVF, in vitro fertilization; ICSI, intracytoplasmic sperm injection; HRT, hormone replacement therapy; hCG, human chorionic gonadotrophin; NA, not available

Regarding the insemination modality of embryo acquisition, the group that recovered from COVID-19 within 6–12 months was more likely to undergo in vitro fertilization than the control group (85.7% vs. 76.3%, *p* = 0.016). Those who recovered from COVID-19 within 6 months (61.7% vs. 81.1%, *p* < 0.001) and 6–12 months (61.7% vs. 80.9%, *p* < 0.001) were more likely to undergo hormone replacement therapy cycles as an endometrial preparation protocol for FET.

HCG positivity, clinical pregnancy, abortion, ongoing pregnancy, and live birth rates were not significantly different between the control group and those who recovered from COVID-19 within 6 or 6–12 months. However, patients who recovered from COVID-19 within 6 months were more likely to experience complications during pregnancy than those in the control group (33.7% vs. 20.3%, *p* = 0.001).

In the multivariate logistic regression analysis of the control group and the group that recovered from COVID-19 within the last 6 months, age ≥ 35 years, transfer of two embryos, and transfer of blastocysts were independent factors influencing the clinical pregnancy rate in the FET cycles. Age ≥ 35 years was a predictor of ongoing pregnancy rate. A history of COVID-19 within the last 6 months was a predictor of pregnancy complication rate (Table 2). Regarding the control group and the group that recovered from COVID-19 within 6–12 months, age ≥ 35 years, natural cycle for endometrial preparation protocol, and transfer of two embryos were independent factors influencing the clinical pregnancy rate for the FET cycles (Table 3).

**Table 2:** Multivariate logistic regression analysis identifying risk factors for clinical and ongoing pregnancy and pregnancy complications post-FET.

	Clinical pregnancy rate		Ongoing pregnancy rate		Pregnancy complications rate	
	*p*-value	aOR (95% CI)	*p*-value	aOR (95% CI)	*p*-value	aOR (95% CI)
**COVID-19 status**						
Recovery from COVID-19 within 6 months	0.599	1.12 (0.73–1.71)	0.714	1.08 (0.71–1.64)	0.013	2.34 (1.19–4.58)
**Age, y**						
≥35	<0.001	0.40 (0.26–0.61)	0.018	0.60 (0.39–0.92)	0.993	1.00 (0.47–2.14)
**BMI**	0.868	1.00 (0.95–1.06)	0.059	0.95 (0.90–1.00)	0.679	0.98 (0.91–1.06)
**Basic FSH**	0.766	0.99 (0.90–1.08)	0.412	0.96 (0.88–1.06)	0.710	1.03 (0.87–1.22)
**Endometrial preparation program**						
Stimulated cycle	0.210	NA	0.085	NA	0.912	
Natural cycle	0.311	1.53 (0.67–3.45)	0.600	1.24 (0.56–2.75)	0.977	1.02 (0.25–4.21)
Hormonal replacement cycle	0.888	1.06 (0.49–2.28)	0.526	0.78 (0.37–1.67)	0.825	1.17 (0.30–4.52)
**Developmental stage of the embryos transferred**						
Blastocyst stage	0.007	1.72 (1.16–2.54)	0.928	1.02 (0.69–1.51)	0.554	1.22 (0.63–2.34)
**Number of embryos transferred**						
2	0.070	1.72 (1.16–2.57)	0.057	1.45 (0.99–2.14)	0.048	1.92 (1.01–3.67)

FET, frozen-thawed embryo transfer; aOR, adjusted odds ratios; CI, confidence interval; COVID-19, coronavirus disease; BMI, body mass index; FSH, follicle-stimulating hormone

**Table 3:** Multivariate logistic regression analysis for identifying predictors of HCG positivity, abortion, and live birth rates after recovery from COVID-19 within 6–12 months.

	Positive HCG rate		Clinic rate	
	*p*-value	aOR (95% CI)	*p*-value	aOR (95% CI)
**COVID-19 status**				
Recovery from COVID-19 within 6–12 months	0.059	1.49 (0.99–2.25)	0.080	1.43 (0.96–2.14)
**Age**				
≥35 years	<0.001	0.35 (0.23–0.54)	<0.001	0.40 (0.26–0.62)
**BMI**	0.407	1.02 (0.97–1.08)	0.776	1.01 (0.96–1.06)
**Basic FSH**	0.327	0.96 (0.88–1.04)	0.398	0.97 (0.89–1.05)
**Infertility type**				
Second infertility	0.569	0.90 (0.63–1.30)	0.449	0.87 (0.61–1.25)
**Fertilization method**				
IVF	NA		0.658	
ICSI	0.425	0.83 (0.52–1.32)	0.419	0.83 (0.53–1.31)
IVF+ICSI	0.747	0.84 (0.28–2.48)	0.718	1.22 (0.42–3.56)
**Endometrial preparation program**				
Stimulated cycle	0.049		0.073	
Natural cycle	0.014	2.65 (1.21–5.77)	0.037	2.27 (1.05–4.91)
Hormonal replacement cycle	0.030	2.22 (1.08–4.59)	0.203	1.60 (0.78–3.27)
**Number of embryos transferred**				
2	<0.001	2.00 (1.35–2.97)	0.003	1.78 (1.22–2.62)

HCG, human chorionic gonadotrophin; aOR, adjusted odds ratio; CI, confidence interval; COVID-19, coronavirus disease; BMI, body mass index; FSH, follicle-stimulating hormone; IVF, in vitro fertilization; ICSI, intracytoplasmic sperm injection.

Regarding the control group and the group that recovered from COVID-19 within the last 6 months, age ≥ 35 years, transfer of two embryos, and transfer of blastocysts were independent factors influencing the HCG positivity rate in the FET cycles. A high body mass index was associated with increased odds of abortion. Age ≥ 35 years, transfer of two embryos, and transfer of blastocysts were independent factors influencing the live birth rate in FET cycles (Table 4). Concerning the control group and the group that recovered from COVID-19 within 6–12 months, age ≥ 35 years, natural cycle for endometrial preparation protocol, hormone replacement therapy cycles for endometrial preparation, and transfer of two embryos were independent factors influencing the positive HCG rate in the FET cycles (Table 3).

**Table 4:** Multivariate logistic regression analysis for identifying predictors of HCG positivity, abortion, and live birth rates after recovery from COVID-19 within 6 months.

	HCG positivity rate		Abortion rate		Live birth rate	
	*p*-value	aOR (95% CI)	*p*-value	aOR (95% CI)	*p*-value	aOR (95% CI)
**COVID-19 status**						
Recovery from COVID-19 within 6 months	0.675	1.10 (0.71–1.69)	0.625	0.83 (0.39–1.77)	0.708	1.09 (0.71–1.66)
**Age**						
≥35 years	<0.001	0.36 (0.24–0.55)	0.478	1.35 (0.59–3.13)	<0.001	0.43 (0.27–0.67)
**BMI**	0.696	1.00 (0.96–1.07)	0.035	1.09 (1.01–1.18)	0.214	0.97 (0.92–1.02)
**Basic FSH**	0.613	0.98 (0.89–1.07)	0.844	0.98 (0.81–1.19)	0.887	0.99 (0.91–1.09)
**Endometrial preparation protocol**						
Stimulated cycle	0.299	NA	0.244	NA	0.094	NA
Natural cycle	0.121	1.92 (0.84–4.37)	0.561	1.90 (0.22–16.53)	0.552	1.28 (0.57–2.90)
Hormonal replacement cycle	0.205	1.65 (0.76–3.58)	0.766	0.89 (0.42–1.89)	0.590	0.81 (0.37–1.75)
**Developmental stage of embryos transferred**						
Blastocyst stage	0.012	1.68 (1.12–2.53)	0.269	3.27 (0.40–26.68)	0.026	1.58 (1.06–2.37)
**Number of embryos transferred**						
2	0.004	1.80 (1.20–2.69)	0.010	0.38 (0.18–0.79)	<0.001	2.02 (1.35–3.00)

HCG, human chorionic gonadotropin; aOR, adjusted odds ratio; CI, confidence interval; COVID-19, coronavirus disease; BMI, body mass index; FSH, follicle-stimulating hormone.

## Discussion

This study compared early and late pregnancy outcomes between patients who underwent FET without COVID-19 and those who recovered from COVID-19. We attempted to confirm whether a history of COVID-19 negatively affected ART outcomes. No significant differences were found in HCG positivity, clinical pregnancy, miscarriage, ongoing pregnancy, and live birth rates; however, a history of COVID-19 within 6 months was an independent predictor of the pregnancy complication rate.

Previous studies have reported the absence of differences in the early outcome measures of fresh or frozen embryo transfer after COVID-19 infection compared to those without infection [8–11]. However, most research did not compare the recovery time after infection; thus, they did not determine the safety of FET during short recovery periods. One study used 60 days after viral infection as a demarcation and found that the clinical and ongoing pregnancy rates within 60 days after FET were lower in the infected group than in the uninfected group, with no difference over 60 days; however, the sample size was too small (n = 29) [6]. Another study [12] conducted FET within the month after recovery from COVID-19 infection and found that positive pregnancy test, implantation, clinical pregnancy, early pregnancy loss, and ongoing pregnancy rates were similar to those in the uninfected group, but the pregnancy outcome was not reported. Thus, maternal and infant safety remains to be demonstrated. To achieve a sufficient sample size and compare the recovery time according to the pregnancy outcome, we employed infection recovery times based on 6 months and still obtained a similar conclusion. This further proves that COVID-19 infection after FET does not significantly affect HCG positivity, clinical pregnancy, and ongoing pregnancy rates.

Several studies have confirmed that pregnant women with SARS-CoV-2 infection have an increased risk of adverse pregnancy outcomes, such as pre-eclampsia, gestational diabetes and premature rupture of membranes, compared with patients without SARS-CoV-2 infection [13–15] and that pregnancy exacerbates COVID-19 symptoms [16–18]. At present, there are few studies on the live birth rate and pregnancy complications of FET after COVID-19 infection; therefore, the live birth rate and pregnancy safety of convalescent FET after COVID-19 infection were key focus points in our study. To control for confounding effects in the regression analysis, we included independent variables that showed significant differences in the univariate analysis as well as factors confirmed to affect treatment outcomes in clinical practice, such as age, body mass index, basal FSH, number of embryo transfers, and endometrial preparation protocol. These factors were entered into the regression analysis as covariates to control for confounding effects. Although the live birth rate was similar to that in uninfected patients, the occurrence of complications during pregnancy requires attention. Our findings confirmed that the incidence of pregnancy complications in patients recovering from COVID-19 within 6 months was 2.34 times greater than that in uninfected patients. Previous studies have found that patients infected with SARS-CoV-2 usually experience various coagulation-related laboratory abnormalities, such as increased platelet activation and aggregation, increased expression of platelet adhesion protein P-selectin, and changes in gene expression in multiple pathways [19,20]. This study reports the highest prevalence of gestational diabetes mellitus and hypertension. A multinational cohort study confirmed that women infected with COVID-19 had a higher risk of pre-eclampsia and eclampsia during pregnancy (RR = 1.76) [17]. In a case report of mid-pregnancy complications of pre-eclampsia with placental abruption, pathology revealed that a novel coronavirus had invaded the placental tissue, providing evidence that the new coronavirus can cause abnormalities in placental function [21]. It has been established that significant hyperglycemia occurs in patients with acute inflammation due to the novel coronavirus (SARS-CoV-2) infection. The presence of the novel coronavirus has been demonstrated to induce abnormal blood glucose levels, resulting in severe insulin resistance and hyperglycemia [22,23]. A study also suggested that although ART may aggravate adverse pregnancy outcomes, it is not a major factor [24]. In this study, all transplanted embryos of the patients were free from viral infection. It can be speculated that COVID-19 infection early before pregnancy can still damage various organs and impair cardiovascular function and metabolic abnormalities. Pregnancy itself confers special immunity, and infection with SARS-CoV-2 may disrupt the balance of this immunity and adversely affect pregnancy. Finally, it is hypothesized that the potential absence of adequate pregnancy care guidelines for expectant mothers during the epidemic period may have contributed to the elevated rate of pregnancy complications [25].

Therefore, after being diagnosed with the novel coronavirus infection, an infertile patient should suspend the embryo transfer process. Before resuming the thawed embryo transfer process, the patient’s recovery should be confirmed. If the patient urgently requests a transfer within 6 months after recovery, the patient should be persuaded to postpone the transfer from the perspective of pregnancy safety. If the transfer is scheduled for 6–12 months after recovery, the patient should be informed of the risks, and antenatal monitoring should be intensified after confirmation of clinical pregnancy until delivery.

The uniqueness of our study lies in its focus on long-term pregnancy outcomes after FET following COVID-19. However, this study had some limitations. First, the analysis of complications in pregnancy was limited by its single-center design and sample size, especially the small number of cases for each type of subdivided complication. This led us to employ a combined analysis strategy, which may have affected the identification of risk factors for specific complications. A stratified analysis is recommended for subsequent multicenter studies after accumulating a sufficient sample size. Second, the study was retrospective; therefore, the severity of infection among the study participants was not stratified. However, to the best of our knowledge, no severe cases of infection have been reported in these patients. In addition, this study did not determine whether the patients had a history of receiving the COVID-19 vaccine.

## Conclusions

This study demonstrated that FET can be conventionally performed during the recovery period after COVID-19. However, enhanced monitoring and follow-up during pregnancy are necessary to ensure the safety of the pregnancy and delivery process. Despite the widespread outbreak of COVID-19, it has not yet stopped spreading. Therefore, the impact of COVID-19 on pregnancy continues to be the subject of ongoing research.

## Supporting information

S1 Table Definition of categorical covariates. (DOCX)

S2 Table Occurrence of various complications in the two groups during pregnancy. (DOCX)

S1 Data Raw data. (XLSX)
