# Supplementary material for: Effects of COVID-19 convalescence on pregnancy outcomes in frozen-thawed embryo transfer: A retrospective cohort study
Source: PLoS One. 2025 Jul 1;20(7):e0326155. doi: 10.1371/journal.pone.0326155 (PMC12212481; doi:10.1371/journal.pone.0326155)
Supplement: S1 Table — (DOCX) [file pone.0326155.s001.docx]

**S1 Table.** Definition of categorical covariates.

| **Covariates** | **Definition** |
| --- | --- |
| Age | < 35 years = 0  ≥35 years old = 1B35 years old = 1 |
| BMI | Body mass index |
| FSH | Follicle-stimulating hormone level |
| Infertility type | Primary infertility: Never been pregnant = 0  Second infertility: She had been pregnant before = 1 |
| Infertility cause | Female factor = 1  Male factor = 2  Both factor = 3  Unexplained infertility = 4 |
| Fertilization method | IVF: in vitro fertilization = 1  ICSI: intracytoplasmic sperm injection = 2  IVF+ICSI = 3 |
| Endometrial preparation program | Stimulated cycle = 1  Natural cycle = 2  Hormonal replacement cycle = 3 |
| Number of embryos transferred | Transplant a single embryo = 1  Transplant two or more embryos = 2 |
| Developmental stage of the embryos transferred | Cleavage stage: cultured in vitro to day 3 = 0  Blastocyst stage: cultured in vitro to day 5 or 6 = 1 |
