# Supplementary material for: Effects of COVID-19 convalescence on pregnancy outcomes in frozen-thawed embryo transfer: A retrospective cohort study
Source: PLoS One. 2025 Jul 1;20(7):e0326155. doi: 10.1371/journal.pone.0326155 (PMC12212481; doi:10.1371/journal.pone.0326155)
Supplement: S2 Table — (DOCX) [file pone.0326155.s002.docx]

**S2 Table.** **Occurrence of various complications in the two groups during pregnancy**

|  | Placenta previa | Diabetes | Hypertension | Premature rupture of membranes | Intrahepatic cholestasis |
| --- | --- | --- | --- | --- | --- |
| Patients without COVID-19 | 2 | 20 | 11 | 2 | 3 |
| Recovery from COVID-19 within 6 months |  | 18 | 11 | 1 | 3 |
